# Members of the Chromobox Family Have Prognostic Value in Hepatocellular Carcinoma

**DOI:** 10.3389/fgene.2022.887925

**Published:** 2022-05-23

**Authors:** Chenxi Pan, Nan Luo, Kun Guo, Wenbo Wang, Lei Li, Ning Fan, Yu Tian

**Affiliations:** ^1^ Department of Breast Surgery, The Second Hospital of Dalian Medical University, Dalian, China; ^2^ Department of Infection, The Second Hospital of Dalian Medical University, Dalian, China; ^3^ Department of Pathology, The Second Hospital of Dalian Medical University, Dalian, China; ^4^ Department of Vascular Surgery, The Second Hospital of Dalian Medical University, Dalian, China; ^5^ College of Basic Medical Sciences, Dalian Medical University, Dalian, China

**Keywords:** chromobox family, hepatocellular carcinoma, prognosis, nomogram, immune infiltration

## Abstract

Liver cancer is the fifth most prevalent malignant tumor, while hepatocellular carcinoma represents the most prevalent subtype worldwide. Previous studies have associated the chromobox family, critical components of epigenetic regulatory complexes, with development of many malignancies owing to their role in inhibiting differentiation and promoting proliferation of cancer cells. However, little is known regarding their function in development and progression of hepatocellular carcinoma. In the present study, we analyzed differential expression, prognostic value, immune cell infiltration, and gene pathway enrichment of chromobox family in hepatocellular carcinoma patients. Next, we performed Pearson’s correlation analysis to determine the relationships between chromobox family proteins with tumor-immune infiltration. Results revealed that high expression of CBX1, CBX2, CBX3, CBX6, and CBX8 was associated with poor survival rates of hepatocellular carcinoma patients. These five factors were used to build prognostic gene models using LASSO Cox regression analysis. Results indicated that high expression of CBX2 and CBX3 proteins was significantly associated with poor prognosis for hepatocellular carcinoma patients. The resulting nomogram revealed that CBX3 and T stages were significantly correlated with prognosis of hepatocellular carcinoma patients. Notably, predictive CBX3 was strongly correlated with immune cell infiltration. Furthermore, results from functional enrichment analysis revealed that CBX3 was mainly involved in regulation of methylation of Histone H3-K27. Collectively, these findings suggest that CBX3 could be a biomarker for predicting prognosis of hepatocellular carcinoma patients.

## 1 Introduction

Liver cancer (LC) is the fourth leading cause of cancer-related deaths worldwide (Llovet et al., 2021; Siegel et al., 2021). Hepatocellular carcinoma (HCC) is the second highest cause of cancer-related deaths globally, accounting for 85–90% of all primary liver malignancies (Bosch et al., 2005). Although alpha fetoprotein (AFP) has low specificity as a serologic diagnostic marker for hepatocellular carcinoma, its sensitivity remains relatively high (around 60%) (Trevisani et al., 2001). Previous studies have reported use of tumor stage in HCC prognosis, with curative therapies for early-stage HCC providing a 5-year survival rate of more than 70% (Llovet et al., 2016; Villanueva, 2019). However, most HCC patients are diagnosed during the late stage of disease development. Notably, prognosis of HCC patients has markedly improved due to advancements in surgical procedures coupled with the use of targeted medications. On the other hand, the median survival rate for asymptomatic advanced cases undergoing systemic therapy is 1–1.5 years (Llovet et al., 2008; Kudo et al., 2018). Therefore, early detection, coupled with use of effective therapy, and prognostic assessment of HCC cases are critical to effective management of the disease.

To date, up to eight members of the CBX family have been discovered in the human genome. CBX family proteins can be classified into two categories based on their molecular structure. Each of these can be further stratified into two subgroups, namely the heterochromatin protein 1 (HP1) (which includes CBX1, CBX3, and CBX5) and the polycomb (Pc) (which comprises CBX2, CBX4, CBX6, CBX7, and CBX8) groups. Specifically, the chromobox (CBX) family proteins represents typical members of the Pc group complex that regulate heterochromatin control, gene expression, and developmental programs (Desai and Pethe, 2020). Previous studies have shown that the HP1 group comprises both N-terminal and a C-terminal chromosome structural domains, whereas the PC group only contains a conserved N-terminal chromosome structural domain (Wotton and Merrill, 2007).

Numerous studies have described the role of members of the CBX family in malignancies of the brain and central nervous cancer (Duan et al., 2018), breast cancer (Iqbal et al., 2021), colorectal cancer (Wang et al., 2016), head and neck cancer (Wang et al., 2017) and sarcoma (Zhou et al., 2021). However, information on their function in HCC is dearth. The Most of the studies have focused on single nucleotide polymorphisms of CBX4 and CBX7 and their association with reduced risk of hepatocellular carcinoma (Tan et al., 2019). Previous research evidence demonstrated that CBX 1/2/3/6/8 is an independent predictor of survival HCC patients (Ning et al., 2018), whereas CBX4 was found to stimulate angiogenesis in HCC via its SUMO E3 ligase activity (Li et al., 2014). To date, however, the role of various members of the CBX family in development and progression of HCC remains unknown. Here, we analyzed expression and mutations across various members of the CBX family members in HCC patients, as well as their relationships to clinical indicators, with a view of elucidating their roles in development and progression of the disease.

## 2 Materials and Methods

### 2.1 Gene Expression Profiling Interactive Analysis (GEPIA)

We employed the GEPIA database (http://gepia.cancer-pku.cn/index.html) to analyze differential gene expression between HCC and normal liver tissues, as well as pathological stage and correlative prognosis analyses Comparisons in expression and pathological stages, between HCC and control groups, were calculated using the Student’s t-test.

### 2.2 Survival Analysis

Raw RNA-sequencing data (level 3) and corresponding clinical information for liver cancer patients were obtained from The Cancer Genome Atlas (TCGA) dataset (https://portal.gdc.cancer.gov/). Acquisition and application of the datasets were performed in accordance with the guidelines and policies. Next, we performed Kaplan-Meier (KM) survival analysis, with log-rank test, to compare differences in the survival between the two groups. *p*-values and hazard ratios (HR), with 95% confidence interval (CI), were generated by log-rank tests and univariate Cox proportional hazards regression. All analyses were performed using packages implemented in R software version v4.0.3 (The R Foundation for Statistical Computing, 2020), and data followed by *p* < 0.05 considered statistically significant.

### 2.3 Development of CBXs Prognostic Signature

The raw counts of RNA-sequencing data (level 3) and accompanying clinical data from HCC patients were collected from TCGA dataset (https://portal.gdc.cancer.gov/) in accordance with the rules and regulations. Additionally, the KM survival analysis combined with the log-rank test was utilized to examine the survival difference between two or more groups. To assess the prediction accuracy of each gene and risk score, a time receiver operating characteristic (ROC) (v 0.4) analysis was done. The above study employs the least absolute shrinkage and selection operator (LASSO) regression approach for feature selection, as well as 10-fold cross-validation (v 4.1–1). LASSO is a well-known machine learning method that has been widely used in medical research (Liu et al., 2021; Liu et al., 2022a; Liu et al., 2022b; Liu et al., 2022c; Liu et al., 2022d).

For Kaplan–Meier curves, log-rank tests and univariate Cox proportional hazards regression were used to provide *p*-values and HR with 95 CI. All analytical procedures and R packages described above were carried out using R version 4.0.3. (The R Foundation for Statistical Computing, 2020). Statistical significance was defined as *p* < 0.05.

Univariate and multivariate cox regression analyses were performed to identify the optimal terms for building the nomogram. We also generated a forest plot to show the *p* value, HR and 95% CI of each variable using the “forestplot” package in R. Results from multivariate Cox proportional hazards analysis were used to develop the nomogram and predict the X-year overall recurrence. The nomogram provided a graphical representation of the factors, which were subsequently used to calculate the risk of recurrence for an individual patient by the points associated with each risk factor using the “rms” package in R.

### 2.4 Correlation Between CBX3 Expression and Immune Infiltration

We employed the TIMER database (https://cistrome.shinyapps.io/timer/), a user-friendly web interface that contains six primary analytical modules, for systematic evaluation of immune cell infiltration and clinical impact. We chose CBX3 as the input, using the “Gene module,” then generated scatterplots to visualize the relationship between its expression and the level of immune infiltration in LC.

### 2.5 The Human Protein Atlas

We utilized the single cell dataset from The Human Protein Atlas (HPA; http://www.proteinatlas.org/) database to determine expression patterns of CBX3 in HCC RNAseq data for CBX3 were utilized to cluster genes, based on expression levels of distinct samples, then the generated clusters manually annotated to describe common aspects in terms of function and specificity. Cluster annotation was combined with confidence scores assigned to the gene of that cluster. The confidence score was calculated as the number of times that gene was allocated to that cluster in a repeated count, and was represented by a value between 0 and 1, where 1 is the greatest attainable confidence level. Clustering results were presented in a Uniform Manifold Approximation and Projection (UMAP), with each cluster emphasizing a colored zone in which most of the grouped genes were allocated.

Next, we determined expression patterns of CBX3 mRNAs and proteins using cell cycle-dependent tests by labeling U-2 OS FUCCI (fluorescence-inactivated cell cycle indicator) cell lines, computational models of cell cycle location, or localization to cell cycle-dependent compartments.

### 2.6 Analysis of Protein-Protein Interaction (PPI)

We utilized GeneMANIA (http://www.genemania.org), which uses a high-precision prediction algorithm to evaluate gene lists and prioritize genes for functional testing, to identify variables that show CBX3’s predictive value. Also, we employed the STRING database (https://string-db.org/) to generate a protein-protein interaction (PPI) network targeting CBX3. Next, we performed analysis of Gene Ontology (GO) terms for CBX3 using STRING’s analysis module.

### 2.7 Oncomine

Expression profiles of mRNAs of distinct members of the CBX family across various cancer types were determined via the Oncomine database (www.oncomine.org), a publicly accessible online tool that allows for genome-wide expression using microarray cancer datasets. In this study, *p* < 0.05, a fold change of 2, and a gene rank in the top 10% were considered statistically significant thresholds. Differences in CBXs expression between two groups in LC were determined using a Student’s t-test.

### 2.8 cBioPortal

Genetic changes and analysis of the network module for CBXs from cBioPortal (www.cbioportal.org) were analyzed via the TCGA database. Next, we employed the survival analysis module to examine variations in overall survival (OS) and disease free survival (DFS) survival rates between CBX3 mutant and unmutated groups.

### 2.9 Genomics of Drug Sensitivity in Cancer

The TCGA dataset (https://portal.gdc.com) was used to retrieve RNA-sequencing expression (level 3) profiles and associated clinical information for HCC. Based on the biggest publicly accessible pharmacogenomics database [the Genomics of Drug Sensitivity in Cancer (GDSC), https://www.cancerrxgene.org/], we predicted the chemotherapeutic response for each sample. The R package “pRRophetic” was used to build the prediction procedure. The half-maximal inhibitory concentration (IC50) of each sample was determined using ridge regression. Every parameter was set to its default value. The batch impact of battle and the tissue type of all tissues were used to calculate the mean value of duplicate gene expression.

The R Foundation for Statistical Computing (2020) version 4.0.3 was used to implement all of the aforementioned analysis techniques and the R package.

## 3 Results

### 3.1 Differential Expression of CBXs in HCC Patients

**FIGURE 1:**
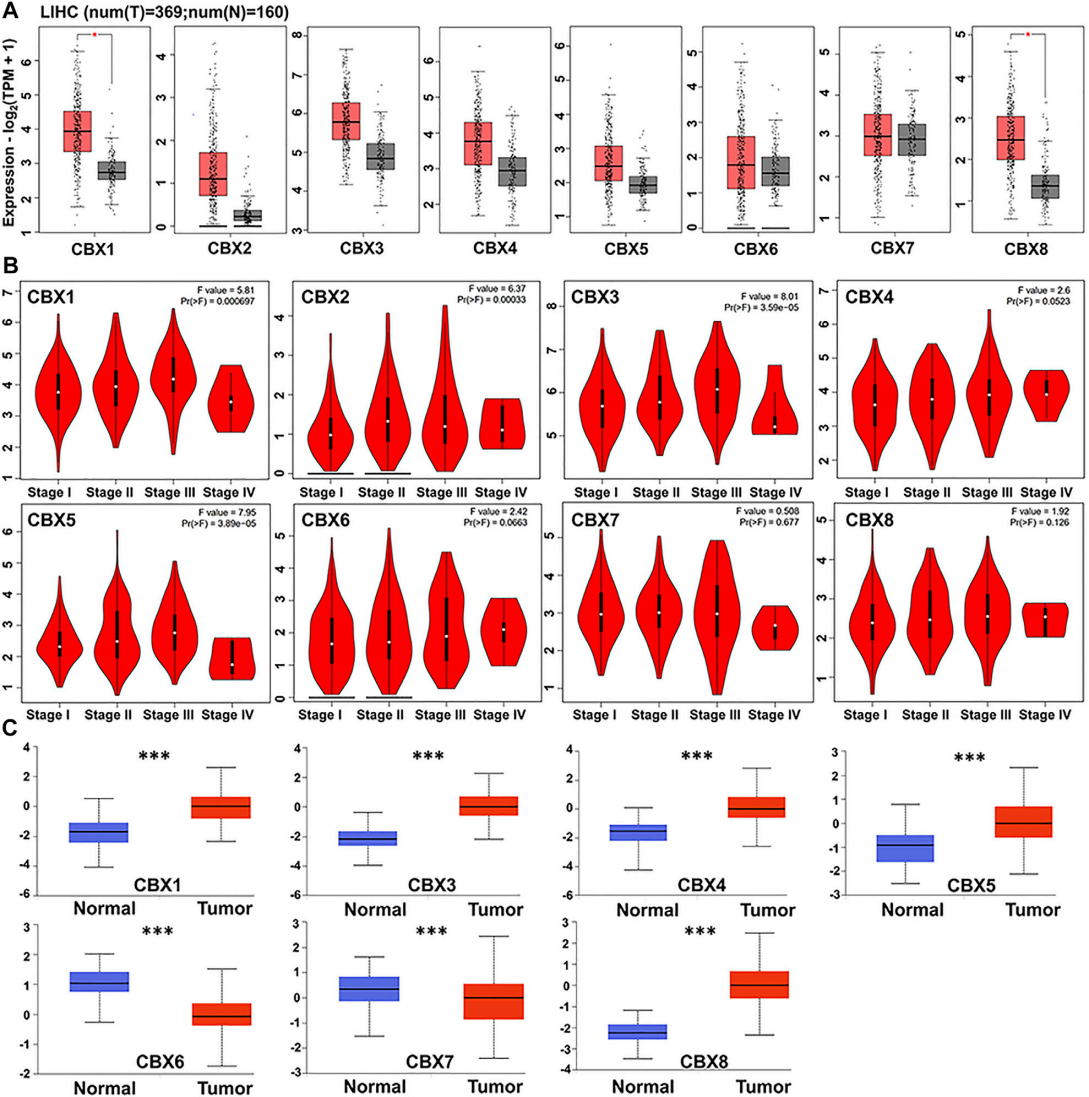
**(A)** CBXs are expressed in HCC (GEPIA). The scatter diagram indicated that CBX1 and CBX8 expression levels were significantly greater in HCC tissues than in normal tissues (*p* < 0.05) **(B)** Correlations between CBX expression and tumor stage in patients with head and neck cancer (GEPIA). CBX1/2/3/5 expression was shown to be significantly linked with the clinical stage of HCC patients (*p* < 0.05). **(C)** Using the CPTAC dataset, we further compared the expression of CBXs total protein in normal and primary HCC tissue. ***p < 0.001.

We used GEPIA to compare expression levels of CBX mRNAs between HCC (n = 369) with normal adjacent liver (n = 160) tissues and found that CBX1 and CBX8 were significantly upregulated in tumor, relative to normal tissues. However, there was no significant change in CBX2-7 expression between HCC and normal tissues (Figure 1A). Next, we examined the relationship between differential CBX expression with clinical stages of HCC patients, and found significant variations in CBX1, CBX2, CBX3, and CBX5 groups, but no significant changes in CBX4, CBX6, CBX7, and CBX8 groups (Figure 1B). These findings suggested that CBX1 may be involved in carcinogenesis and development of HCC. Notably, we found significant differential expression I proteins of all members of the CBX family. Moreover, CBX1/3/4/5/8 was significantly upregulated, whereas CBX6/7 was significantly downregulated in HCC, relative to normal tissues. However, we obtained no data on CBX2 expression (Fig, 1C).

### 3.2 Prognostic Value of CBX Expression in HCC Patients

**FIGURE 2:**
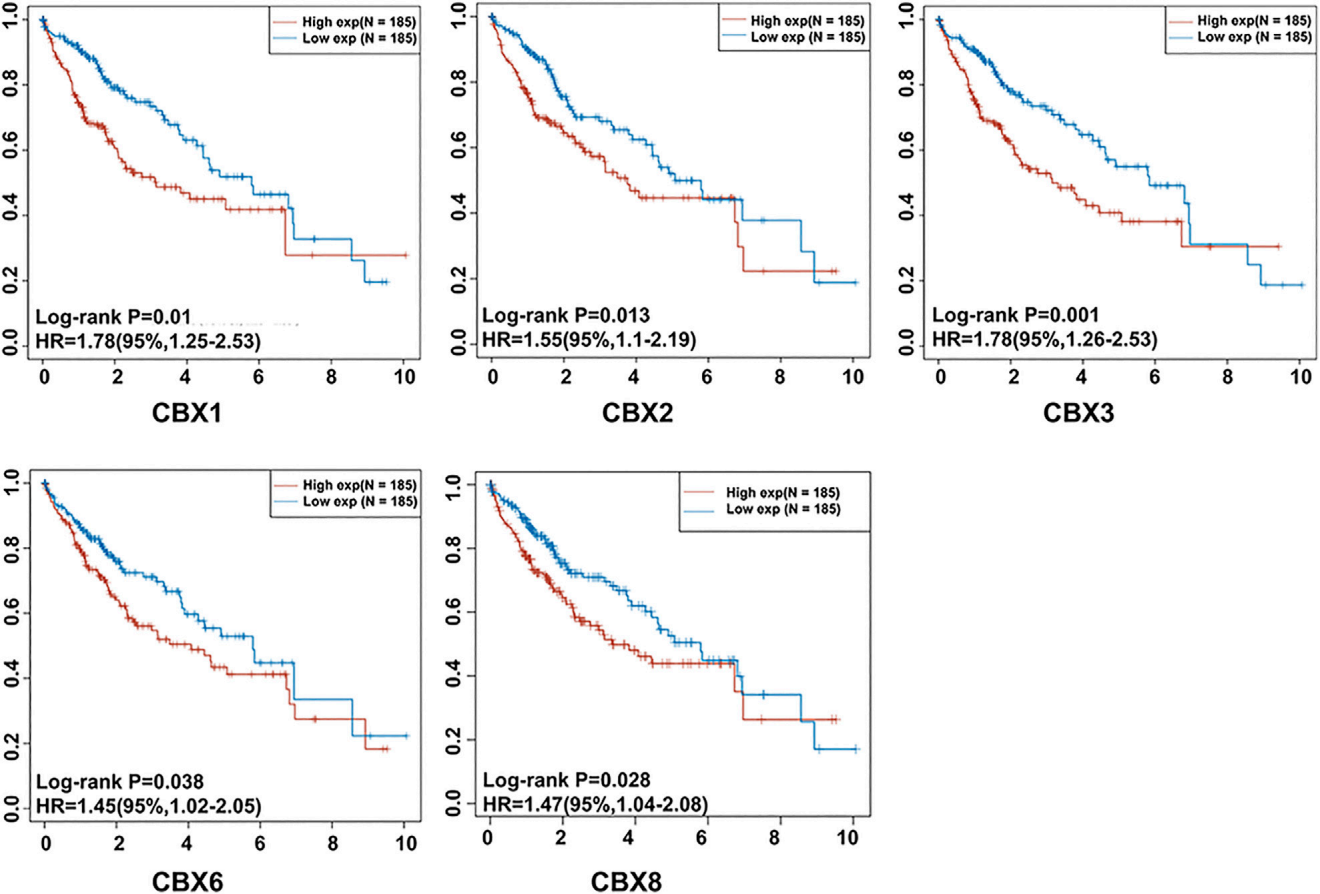
The prognostic significance of unique CBX family members’ mRNA expression in HCC. Patients with HCC who had elevated CBX1/2/3/6/8 transcriptional levels had a substantially shorter OS (*p* < 0.05).

We determined the relationship between mRNA expression of distinct CBXs with clinical outcomes, with a view of understanding their role in HCC progression. A summary of clinical characteristics of TCGA-HCC patients is outlined in Table 1, while the OS curves are depicted in Figure 2. Summarily, expression of CBX1, CBX2, CBX3, CBX6, and CBX8 was significantly associated with OS (CBX1: *p* = 0.001, HR = 1.78; CBX2: *p* = 0.013, HR = 1.55; CBX3: *p* = 0.001, HR = 1.78; CBX6: *p* = 0.038, HR = 1.45; CBX8: *p* = 0.028, HR = 1.47). On the other hand, CBX1/2/3/6/8 overexpression was significantly associated with poor OS of patients.

**TABLE 1:** Clinical information for patients with TCGA-HCC.

	Characteristic	Number
Status	Alive	241
	Dead	130
Age	Mean (SD)	59.4 (13.5)
	Median [MIN, MAX]	61 [16,90]
Gender	FEMALE	121
	MALE	250
Race	AMERICAN INDIAN	2
	ASIAN	158
	BLACK	17
	WHITE	184
pT_stage	T1	181
	T2	92
	T2a	1
	T2b	1
	T3	45
	T3a	29
	T3b	6
	T4	13
	TX	1
pN_stage	N0	252
	N1	4
	NX	114
pM_stage	M0	266
	M1	4
	MX	101
pTNM_stage	I	171
	II	86
	III	3
	IIIA	65
	IIIB	8
	IIIC	9
	IV	2
	IVA	1
	IVB	2
Grade	G1	55
	G2	177
	G3	122
	G4	12
new_tumor_event_type	Primary	10
	Recurrence	163
Radiation_therapy	Non-radiation	240
	Radiation	4
History_of_neoadjuvant_treatment	Neoadjuvant	2
	No neoadjuvant	369
Therapy_type	Ancillary	1
	Chemotherapy	29
	Chemotherapy:Hormone Therapy	1
	Chemotherapy:Hormone Therapy:Other. specify in notes	1

### 3.3 Construction of a CBX-Related Prognostic Model

**FIGURE 3:**
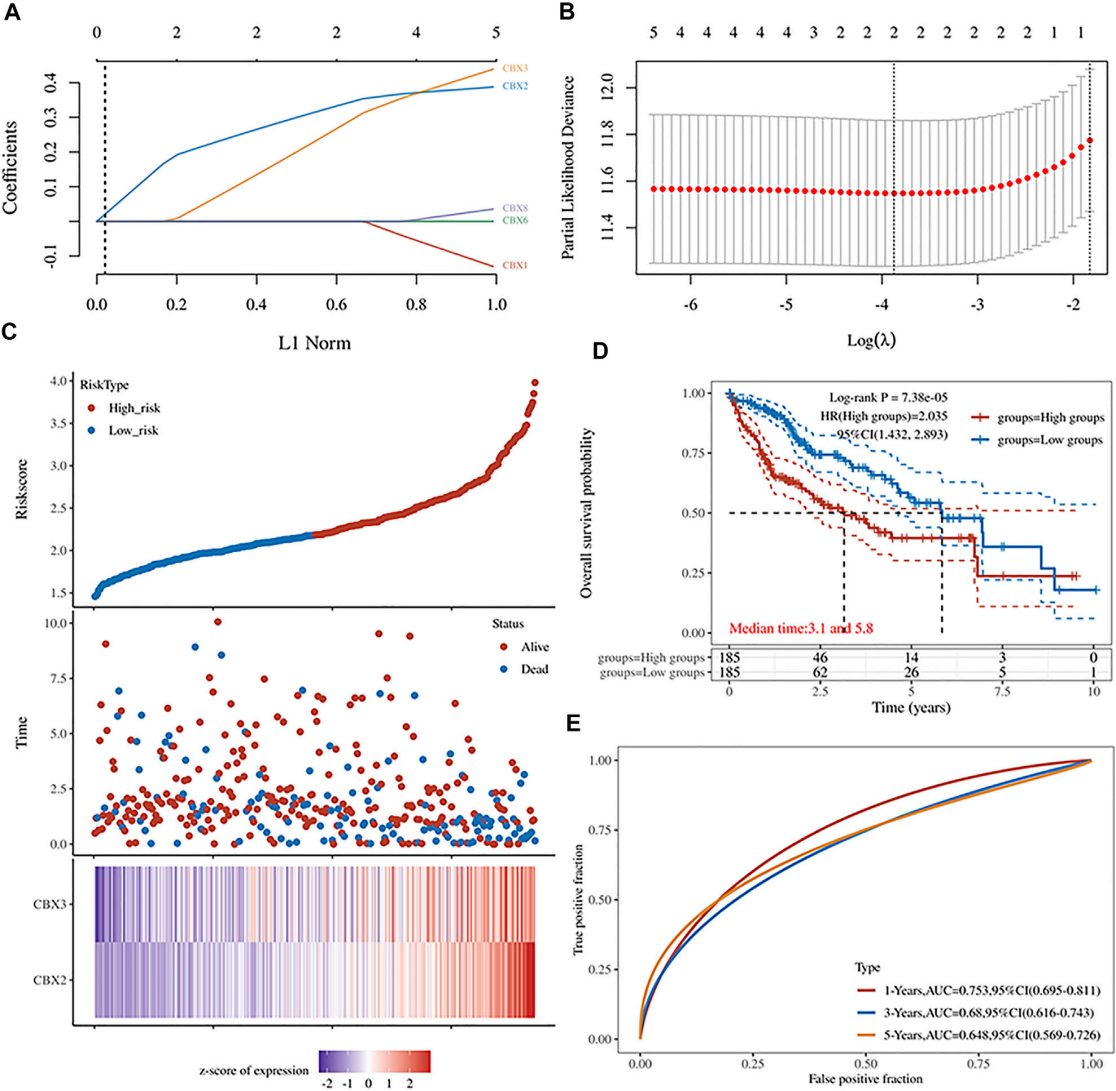
**(A)** The five CBXs’ LASSO coefficient profiles **(B)** Plots of the cross-validation error rates tenfold. **(C)** Risk score distribution, survival status, and expression of five prognostic CBXs in HCC **(D,E)** Overall survival curves for HCC patients classified as high- or low-risk, as well as the receiver operating characteristic (ROC) curve used to determine predictive value.

LASSO is a regression-based methodology that allows for the inclusion of a high number of covariates in the model, and crucially has the unique property of penalizing the absolute magnitude of a regression coefficient, thereby limiting the impact of a coefficient on the entire regression. A higher penalty results in a dramatic drop of coefficients, until it reaches zero, so automatically deleting superfluous/influential variables (Efron et al., 2004; Bickel et al., 2009; Lee et al., 2016). In the present study, we obtained five genes with prognostic significance. Their Kaplan–Meier survival curves are depicted in Figure 2. Next, we incorporated these genes in LASSO Cox regression to develop a prognostic model (Figures 3A,B), and calculated their risk scores as follows (0.3322)∗CBX2+(0.2664)∗CBX3. Next, we used the median risk score to stratify LC patients into two groups. Risk score distribution, survival status, and expression profiles of these two genes are presented in Figure 3C. Interestingly, a higher risk score was associated with an increase in mortality rates and shorter time to death in patients (Figure 3C). Kaplan–Meier curves demonstrated that HCC patients in the high-risk group had significantly lower OS rates than those in the low-risk group (median time = 3.1 vs 5.8 years, *p* = 7.38e-05, Figure 3D). The area under the curves (AUC) of the ROC were 0.753, 0.68, and 0.648 for 1-, 3-, and 5-year survival, respectively (Figure 3E).

### 3.4 Nomogram for Predicting HCC

**FIGURE 4:**
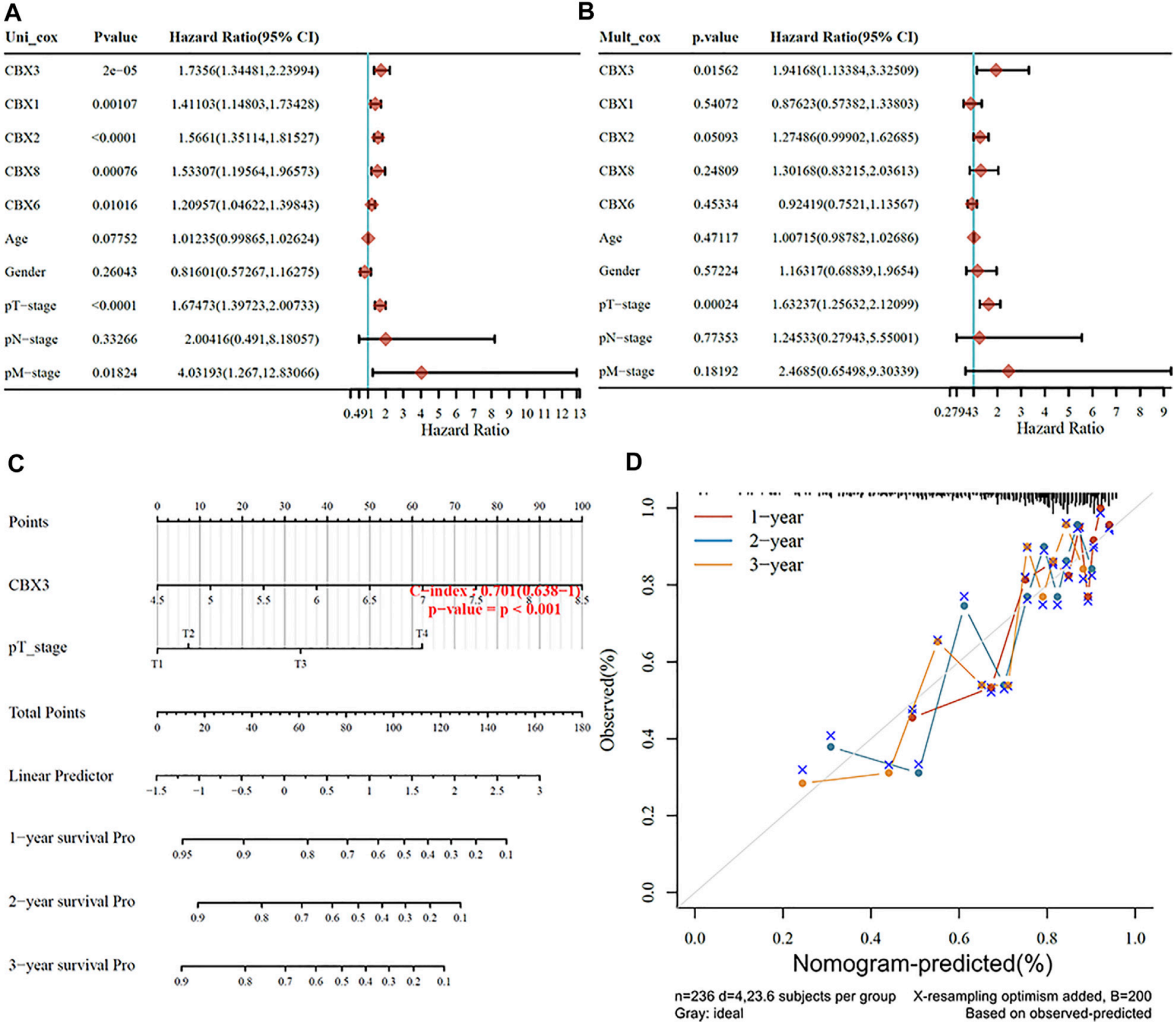
**(A,B)** Hazard ratios and *p* values for the components included in univariate and multivariate Cox regression analyses of HCC clinical variables and five prognostic CBXs. **(C,D)** Nomogram to estimate the overall survival rate of HCC patients at one, three, and 5 years. Calibration curve for the discovery group’s overall survival nomogram model. The ideal nomogram is shown by a dashed diagonal line.

We used patients’ clinicopathologic features and the aforementioned five prognostic CBX genes to build a nomogram for predicting survival of HCC patients. Results from univariate and multivariate analyses revealed that CBX3 expression and pT stage were independent predictors for prognosis of HCC patients (Figures 4A,B). Notably, the nomogram had excellent value in predicting 1-,2- and 3-year overall survival rates of patients, relative to an ideal model in the entire cohort (Figures 4C,D).

### 3.5 Relationship Between CBX3 Expression With Immune Cell Infiltration in HCC Patients

**FIGURE 5:**
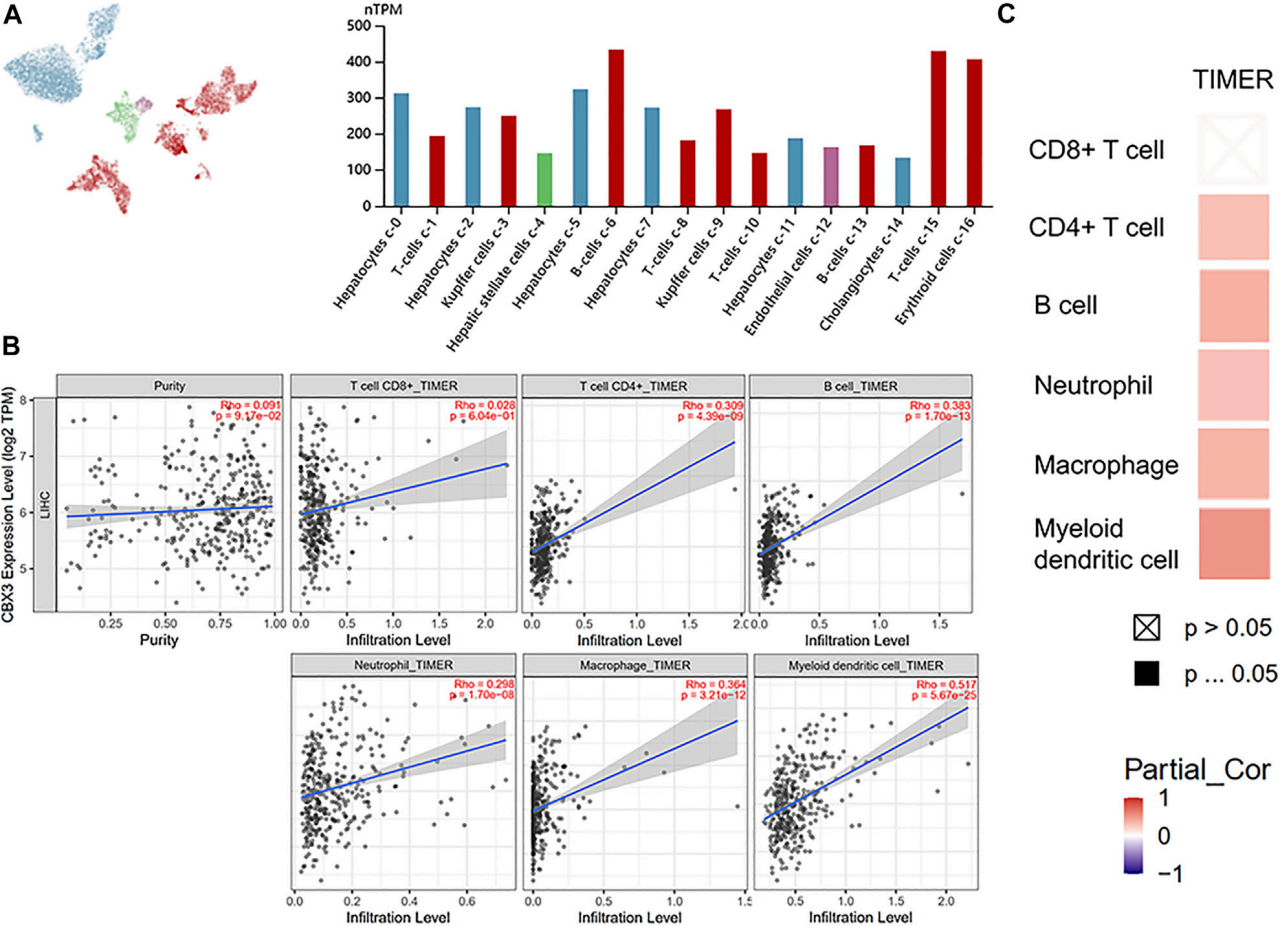
**(A)** CBX3 and liver single cell sequencing results (HPA) **(B)** The relationship between the number of immune cells and CBX3 expression. **(C)** A heatmap depicting the number of immune cells and CBX3 expression.

Results from the HPA database revealed that upregulation of CBX3 was significantly associated with immune cell infiltration, most in B and T cells (Figure 5A). Analysis based on the TIMER database also successfully revealed the relationship between CBX3 expression and immune cell infiltration (Figures 5B,C). Particularly, CBX3 expression was associated with infiltration of CD4^+^ T cells, neutrophils, B cells, macrophages, neutrophils, and dendritic cells in HCC. Results from both databases were generally consistent, indicating that CBX3 expression was closely associated with immune infiltration.

### 3.6 Expression and Interaction Analyses of CBX3

**FIGURE 6:**
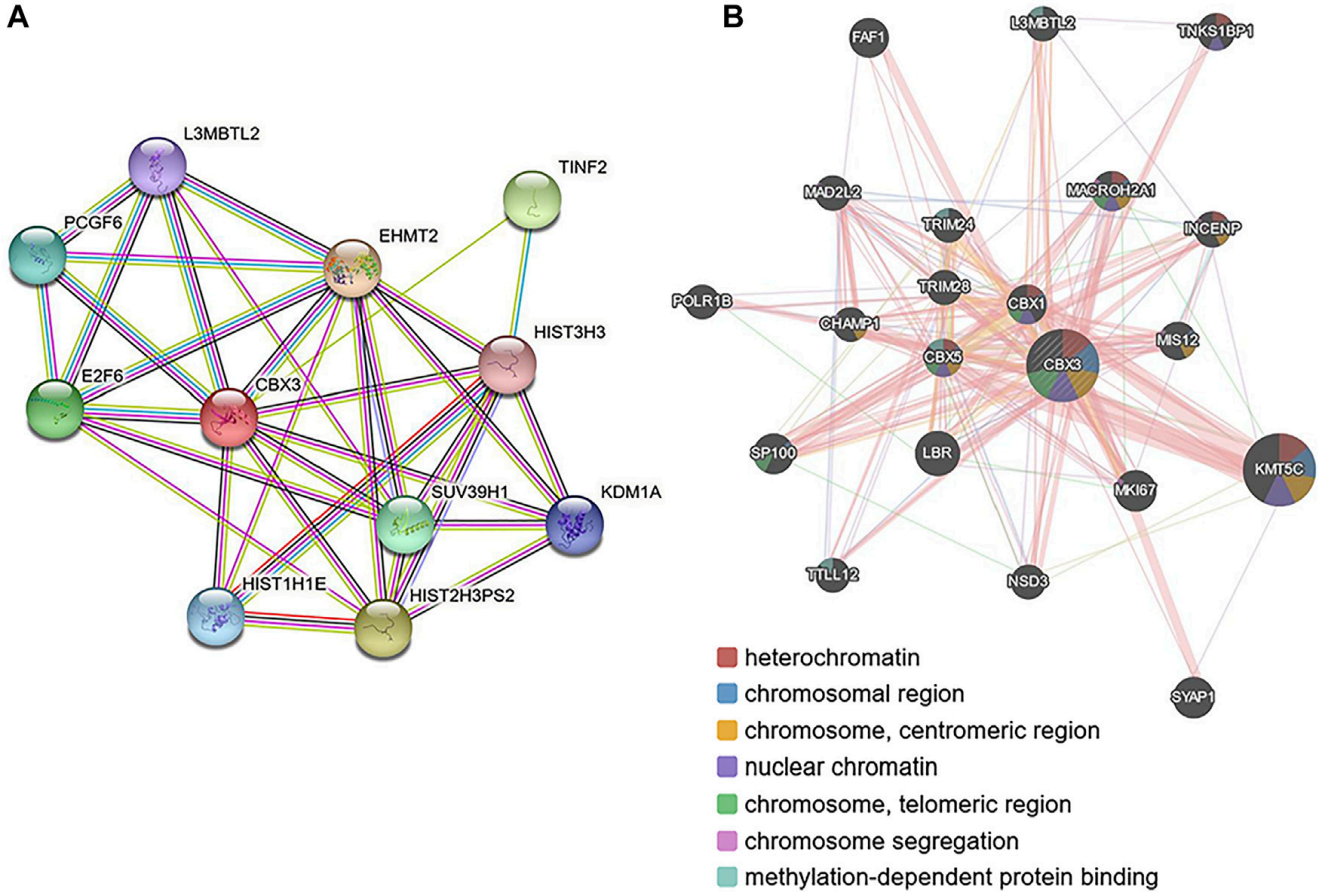
Protein–protein interaction network of different expressed CBX3 **(A)** STRING (https://string-db.org/) **(B)** GeneMANIA (http://www.genemania.org).

The PPI network revealed 11 nodes and 13 edges (*p* = 7.04e-07; Figure 6A). Notably, differential expression of CBX3 was significantly associated with transcriptional regulation by E2F6 (*p* = 1.45e-07). Meanwhile, Gene Ontology terms revealed that CBX3 was mainly involved in Histone H3-K27 methylation (Biological Process: *p* = 0.0132; Molecular Function: *p* = 0.0493). Other GO terms indicated that it also played a role in the nucleus. Results from GeneMANIA showed that the differentially expressed CBX3 and their associated molecules, including KMT5C, LBR, MKI67, SYAP1 and SP100 (Figure 6B), were mainly involved in regulation of heterochromatin, chromosome, nuclear chromatin and methylation-dependent protein binding (Table 2).

**TABLE 2:** CBX3-related GO analysis (STRING).

Cellular component (gene ontology)	Term description	Observed gene count	Background gene count	Strength	False discovery rate
GO:0000228	Nuclear chromosome	8	1,256	1.05	0.0001
GO:0000784	Nuclear chromosome, telomeric region	4	102	1.84	0.00032
GO:0098687	Chromosomal region	5	318	1.45	0.00037
GO:0000785	Chromatin	7	1,220	1.01	0.0004
GO:0031981	Nuclear lumen	10	4,733	0.57	0.0021
GO:0000790	Nuclear chromatin	6	1,048	1.01	0.0027
GO:0000792	Heterochromatin	3	76	1.85	0.0028
GO:0005654	Nucleoplasm	9	3,973	0.61	0.0053
GO:0005719	Nuclear euchromatin	2	31	2.06	0.0263
GO:0032993	protein-DNA complex	3	195	1.44	0.0263
GO:0005720	Nuclear heterochromatin	2	35	2.01	0.029
GO:0000788	Nuclear nucleosome	2	38	1.97	0.032
Molecular Function (Gene Ontology)					
GO:0003682	Chromatin binding	5	570	1.19	0.0448
GO:0046974	Histone methyltransferase activity (h3-k9 specific)	2	10	2.55	0.0493
Biological Process (Gene Ontology)					
GO:0070317	Negative regulation of g0 to g1 transition	5	43	2.32	5.72E-07
GO:0051276	Chromosome organization	9	1,066	1.18	1.28E-06
GO:0006325	Chromatin organization	8	713	1.3	2.13E-06
GO:2000113	Negative regulation of cellular macromolecule biosynthetic process	9	1,470	1.04	1.31E-05
GO:0045934	Negative regulation of nucleobase-containing compound metabolic process	9	1,528	1.02	1.54E-05
GO:0045892	Negative regulation of transcription, dna-templated	8	1,273	1.05	7.96E-05
GO:0016570	Histone modification	5	351	1.4	0.00084
GO:0000122	Negative regulation of transcription by rna polymerase ii	6	895	1.08	0.0028
GO:0034968	Histone lysine methylation	3	71	1.88	0.0061
GO:0070734	Histone h3-k27 methylation	2	11	2.51	0.0132
GO:2001251	Negative regulation of chromosome organization	3	137	1.59	0.0261
GO:0018027	Peptidyl-lysine dimethylation	2	19	2.27	0.0269
GO:0031497	Chromatin assembly	3	172	1.49	0.0437

## 4 Discussion

Controlling gene expression precisely is critical for cell function. Although transcription is ultimately guided by DNA-binding transcription factors, it is also necessary for chromatin and histone post-translational modification. Eight CBX proteins have been found in the human genome, all of which have a similar chemical structure and contain a single N-terminal chromodomain. All of the CBX proteins are involved in heterochromatin control, gene expression, and developmental programs. CBX2, CBX4, CBX6, CBX7, and CBX8 are conventional PRC1 components that cooperate with PRC2 to regulate gene expression. PRC1 CBX protein components identify and bind to H3K27me3 mediated by PRC2, hence recruiting PRC1 to transcriptionally regulated target genes. The remaining three CBX proteins (CBX1, 3, and 5) are referred to as heterochromatin protein 1 (HP1), HP1 (HP1), and HP1 (HP1). They (CBX1, 3, and 5) are heterochromatin protein 1 (HP1) complex-associated methyl readers that decode H3K9me3 marks generated by H3K9 methyltransferases (van Wijnen et al., 2021). Using nonhomologous protein sequences, unique polycomb group CBX proteins were associated with diverse areas of chromatin, implying that each polycomb group protein has a distinctive target. Notably, members of the CBX family have been implicated in the development of a range of malignancies, including HCC (Supplementary Figure S1) (Kulis and Esteller, 2010). Despite the fact that numerous members of the CBX family have been implicated in the development of HCC, the precise functions of others remain uncertain. The primary goal of this work was to examine the expression patterns, prognostic value, and immune infiltration of several CBX family members in HCC and to develop a nomogram model to improve patient prognosis.

Our findings indicated that CBX1 and CBX8 mRNAs were significantly upregulated in HCC tumor, relative to adjacent normal, tissues. Apart from CBX2, whose data was missing, all other CBX family proteins were significantly differentially expressed between tumor and normal tissues in HCC patients. Moreover, CBX1/3/4/5/8 was significantly upregulated in tumor, relative to normal tissues, which was consistent with the trend of expression observed in the TGCA database. The expression pattern for CBX1/3/4/5/8 proteins was also consistent with findings from previous studies (Jiao et al., 2015; Yang et al., 2018; Tang et al., 2019; Zhong et al., 2019). Although expression of CBX6/7 proteins were significantly upregulated in tumor tissues, its mRNA levels were almost similar between tumor and normal tissues based on the TCGA database. There was no consistency between mRNA and protein expression for this factor. Our results on CBX6 protein expression were in contrast to previous findings (Wang et al., 2020a), thus datasets from larger clinical trials are required to validate this phenomenon. Expression of CBX1/2/3/5 mRNA was significantly associated with each patient’s cancer stage, suggesting that it be playing a crucial role in HCC progression. Based on this, we hypothesize that the HP1 subtype plays a direct role in HCC progression. Additionally, CBX4 and CBX8 of the Pc subtype may exert their effects on HCC via post-transcriptional changes such as phosphorylation and sumoylation (Zhan et al., 2018; Wang et al., 2020b). The particular mechanism via which the Pc subtype affects HCC requires experimental confirmation. To summarize, the CBX family was expressed significantly differentially in HCC, and the trend of expression of the eight CBX families were inconsistent. The mechanism by which the CBX family is involved in HCC requires further investigation.

Our results further indicated that expression of members of the CBX family was strongly associated with prognosis of HCC patients. Moreover, expression of CBX1/2/3/6/8 was significantly associated with prognosis of HCC patients, with its upregulation correlated with poor OS rates. Considering the high expression of members of the CBXs family in HCC, we postulate that CBX1/2/3/8 and CBX6 are oncogenes. Results from LASSO regression analysis indicate that CBX2 and CBX3 were more essential for survival of HCC patients than the other five genes. Additionally, CBX2 and CBX3 acted as oncogenes in HCC, as illustrated in Figure 3B. This result was consistent with our preceding conclusion.

There was a significant association between the CBXs family and the prognosis of HCC. Using LASSO regression, we determined that CBX2 and CBX3 were more essential for survival prognosis than the other five genes. Additionally, as illustrated in Figure 3B, CBX2 and CBX3 operate as oncogenes in HCC. This finding is completely consistent with the prior conclusion. The Nomogram supports our hypothesis that CBX3 expression and T-stage together have an effect on the OS prognosis of HCC, that CBX3 has a significantly bigger effect on HCC than T-stage, and that CBX3 will function as an independent prognostic biomarker for HCC. Simultaneously, we validated our results against a variety of other databases (Supplementary Figure S5). However, we discovered no statistically significant relationship between the N and M phases and the prognosis of HCC, which spreads primarily through hematologic metastasis and extremely rarely *via* lymph node metastasis. Although lymph nodes are the second site of extrahepatic metastasis in hepatocellular carcinoma, their rarity in HCC may explain why HCC is not associated with N stage (Liang et al., 2022). Intrahepatic metastasis was the most frequent kind of metastasis in HCC, while extrahepatic metastasis was less frequent. There were four patients with M1 and nine individuals with Mx in TGCA-HCC (Table 1). Inadequate data may skew the outcomes. This study contains several flaws. TGCA-HCC did not include liver function or vascular invasion (Bruix et al., 2016). These two indicators have a strong predictive value for the prognosis of HCC. Investigators should incorporate liver function and vascular invasion in subsequent big clinical trials. This has a stronger bearing on clinical work.

After identifying CBX3 as an independent prognostic factor for HCC, we analyzed its role in patient prognosis. Firstly, we eliminated the idea that CBX3 was mutating and impacting HCC development. We detected CBX3 mutations in 1.6% of the 366 HCC patients, but found no statistically significant differences in outcome between persons with and without CBX3 mutations (Supplementary Figure S2A). Further analysis indicated that CBX3 mutations did not appear to play a role in HCC progression (Supplementary Figure S2B, C). Next, we employed the HPA database to analyze the relationship between CBX3 expression and immune cell infiltration and found that CBX3 was significantly upregulated in immune cells relative to other members of the CBX family (Supplementary Figure S3). Moreover, CBX5 was dramatically elevated in T cells, but remained significantly downregulated relative to CBX3. Secondly, we found that CBX3 was significantly upregulated in immune cells relative to other cell subpopulations, with B cells and T cells being the most abundant. Results from the TIMER database indicated that CBX3 expression was strongly correlated with B- and DC-cell infiltration. Notably, CBX3 most likely affected the microenvironment of HCC tumors *via* B cells, CD4^+^ T cells, and DC cells. Recent research targeting HCC and TME have demonstrated that both CD4^+^ and CD8^+^ T cells possess anticancer potential. In fact, researchers have proved that CD8^+^ T cells and the widely used PD-1 therapy significantly improve prognosis of HCC patients (Iwai et al., 2002), whereas CD4^+^ T cells are also components of activated CD8^+^ T cells (Borst et al., 2018). Collectively, these findings demonstrated the crucial role played by T cells in the immunological treatment of HCC, which has subsequently expanded the use of DC cells in liver immunotherapy. A combination of DC immunization and PD-L1 inhibitors has shown promise as a novel treatment approach for HCC (Teng et al., 2020). In fact, a dose-dependent concentration of this regimen resulted in significantly longer overall survival, decreased tumor volume, and increased tumor cell apoptosis in mice relative to either treatment alone (Teng et al., 2020). This was achieved by inducing a stronger anti-tumor cytotoxic T cell response. Results of the present study indicated that CBX3 expression was strongly correlated with infiltration of DC cells, suggesting that CBX3 may be a key target for future development of DC cells in hepatocellular carcinoma. However, there was a substantial link between CBX3 expression and B cells, although there are diverse opinions regarding the role of B cells in HCC. While B cells have been shown to promote CTL activity through secretion of cytokines, cytokines released by B cells reportedly promote angiogenesis and tumor formation (de Visser et al., 2005; Tsou et al., 2016). Immunotherapy is expected to drastically improve prognosis of HCC patients in the near future.

Since proteins represent the primary mode of execution for CBX family functions, we analyzed expression of proteins known to interact with CBX3. Results indicated that CBX3 has a strong affinity for the CBX and HIST families. Notably, CBX3 was expressed in the nucleus, and is a member of the HP1 subfamily of the CBX family (Supplementary Figure S4A). Previous studies have shown that HP1 proteins directly interact with the methylated H3K9 promoter region, thereby regulating chromatin, gene expression, and growth and development (Lomberk et al., 2006; Bot et al., 2017). Additional research evidences have associated aberrant expression of members of the HP1 group with progression of several human diseases and malignancies (Thorne et al., 2012; Slezak et al., 2013). In the present study, GO terms revealed that CBX3 was not only involved in nuclear chromatin regulation and Histone methyltransferase activity, but also played a role in regulation of the cell cycle (Supplementary Figure S4B). Moreover, CBX3 was not only highly expressed in the G1 phase but also acted as a barrier to cell progression from the G0 to the G1 phase (Supplementary Figure S4C, D). Therefore, CBX3’s unique and central biological functions suggests its potential as a target for development of treatment therapies for HCC patients.

Currently, HCC is mostly treated with three types of drugs. Chemotherapy is predominantly FOLFOX4, targeted treatment is predominantly sorafenib, and immunotherapy is either atenizumab plus bevacizumab or sindilizumab plus bevacizumab. The website GDSC estimated the sensitivity of CBX3^high^ HCC patients to standard chemotherapy and targeted therapies (Supplementary Figure S6). As can be shown in Supplementary Fig. 6D, CBX3^high^ HCC patients were responsive to sorafenib but insensitive to platinum chemotherapeutic treatments (Supplementary Figure S6A). As a result, platinum chemotherapy agents should be considered when treating CBX3^high^ HCC patients with the usual FOLFOX4 regimen. Sorafenib was indicated for CBX3^high^ HCC patients.

In conclusion, CBX3 is an independent prognostic factor for HCC. Functionally, it inhibits development of HCC tumor *via* immune cell infiltration. Overall, these findings indicate that CBX3 is a novel biomarker for hepatocellular carcinoma, and a potential target for future development of therapies for HCC treatment.

## Data Availability

The original contributions presented in the study are included in the article/Supplementary Material, further inquiries can be directed to the corresponding author.
