# Supplementary material for: Members of the Chromobox Family Have Prognostic Value in Hepatocellular Carcinoma
Source: Front Genet. 2022 May 23;13:887925. doi: 10.3389/fgene.2022.887925 (PMC9168656; doi:10.3389/fgene.2022.887925)
Supplement: Supplementary file 1 [file DataSheet1.docx]

Supplementary Material

## Supplementary Figures


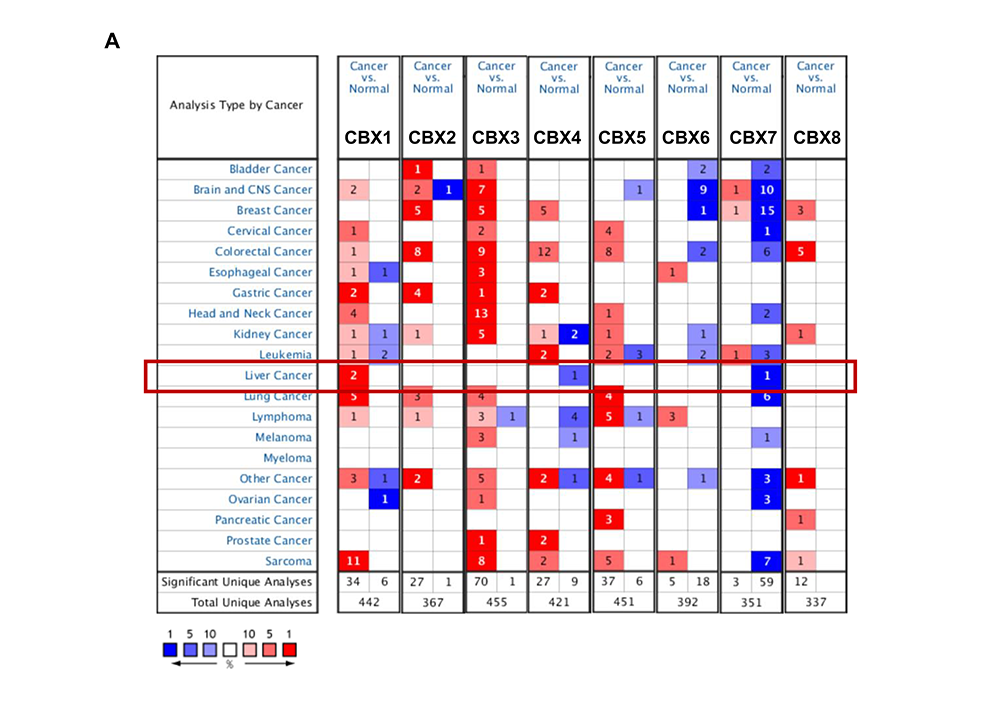


**Supplementary Figure 1.** CBX family members' mRNA expression in various cancer types (Oncomine). The image depicts the number of datasets with statistically significant changes in the target gene's mRNA expression: upregulated (red) and downregulated (blue) (blue). We utilized the following criteria: p-value = 0.01, fold change = 2, gene rank = 10%, data type: messenger RNA, analysis type: cancer vs. normal tissue.

**
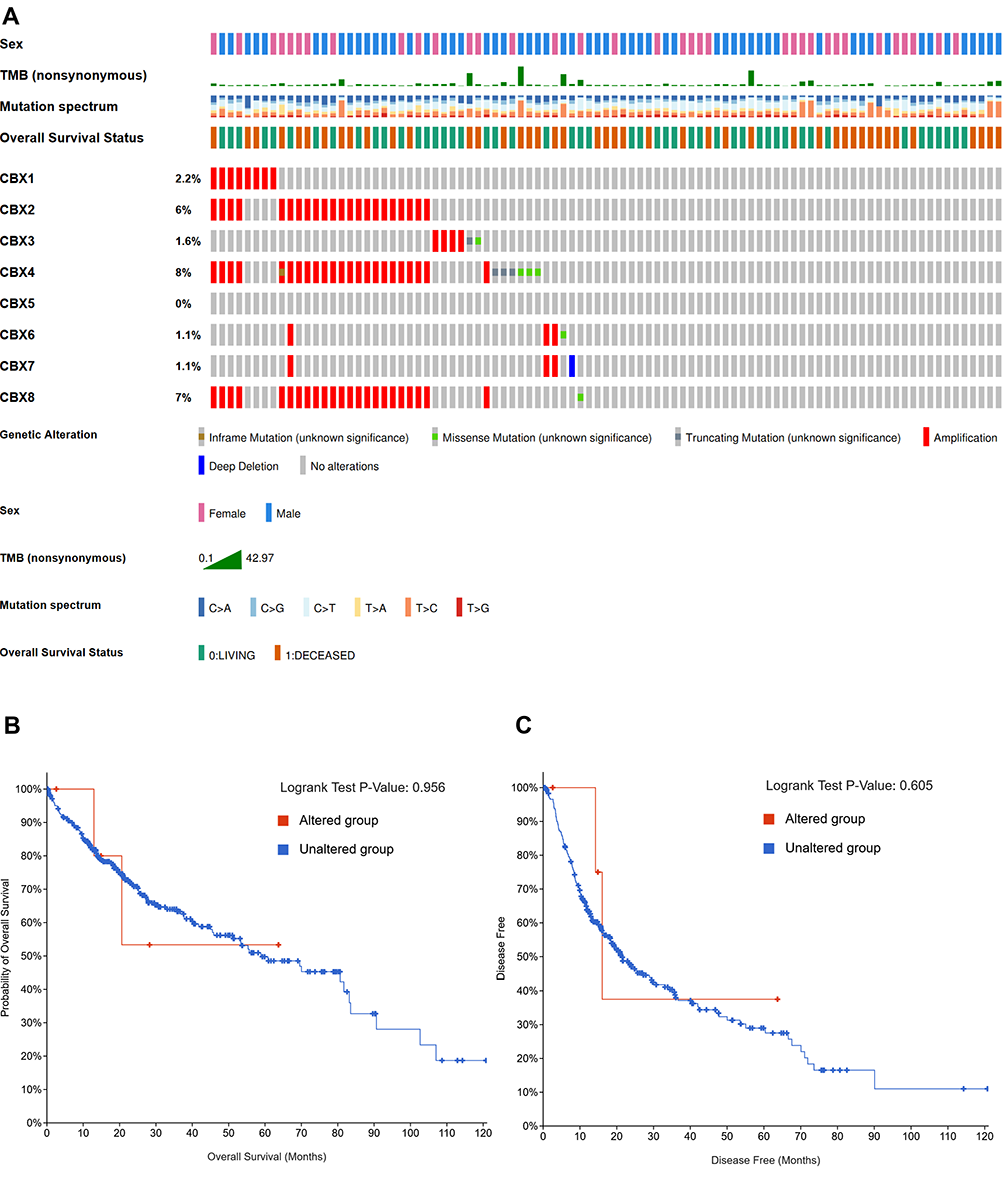
**

**Supplementary Figure 2.** Mutation and expression analysis of the CBX gene in HCC (cBioPortal). (A) A summary of the changes in the expression of several CBXs in HCC. CBXs were changed in 44 samples from 366 HCC patients, accounting for 13% of the samples. The effect of CBX3 mutations on the prognosis of OS (B) and DFS (C).


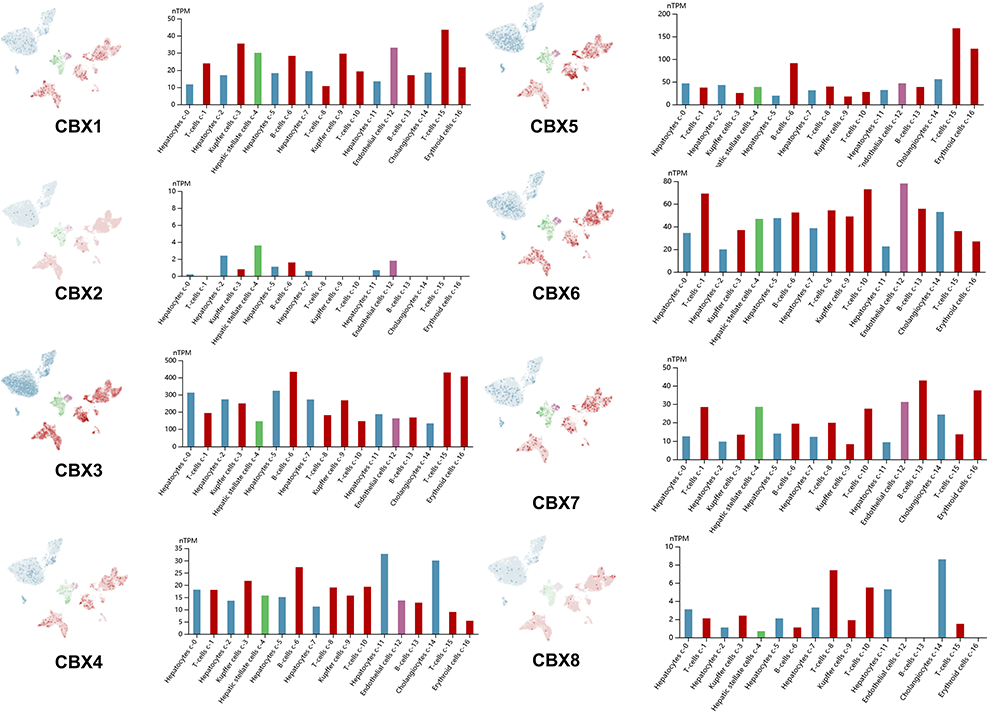


**Supplementary Figure 3.** Results of CBXs and liver single cell sequencing (HPA).


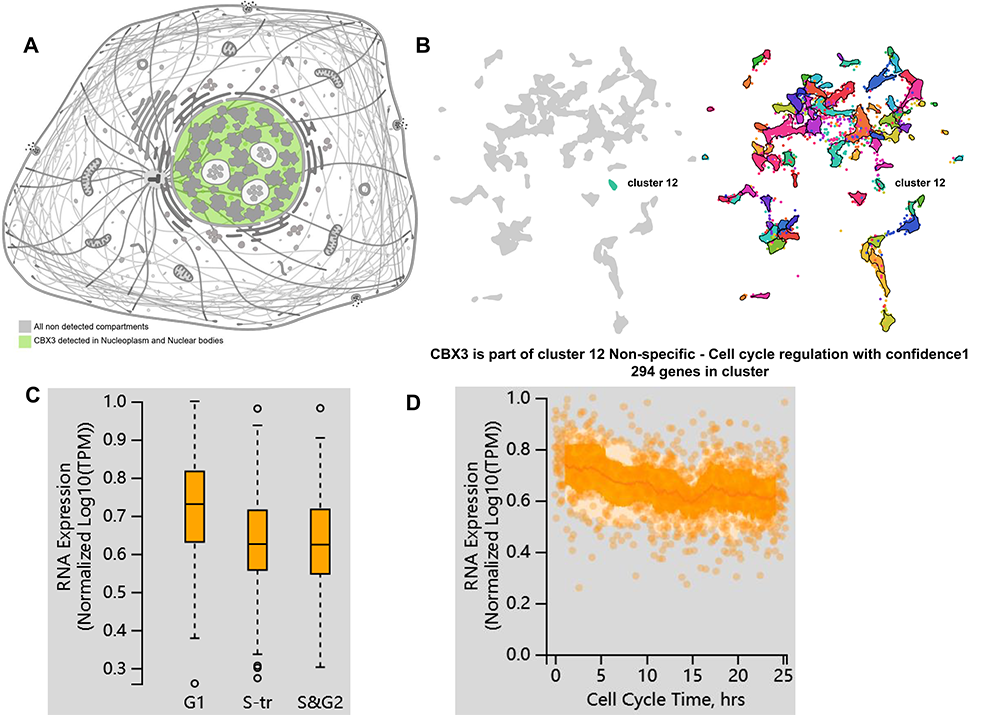


**Supplementary Figure 4.** (A)CBX3 is expressed in the nucleus (HPA). (B) CBX3 is part of cluster 12 non-specific-cell cycle regulation. (C, D) CBX3 RNA expression across Cell cycle.


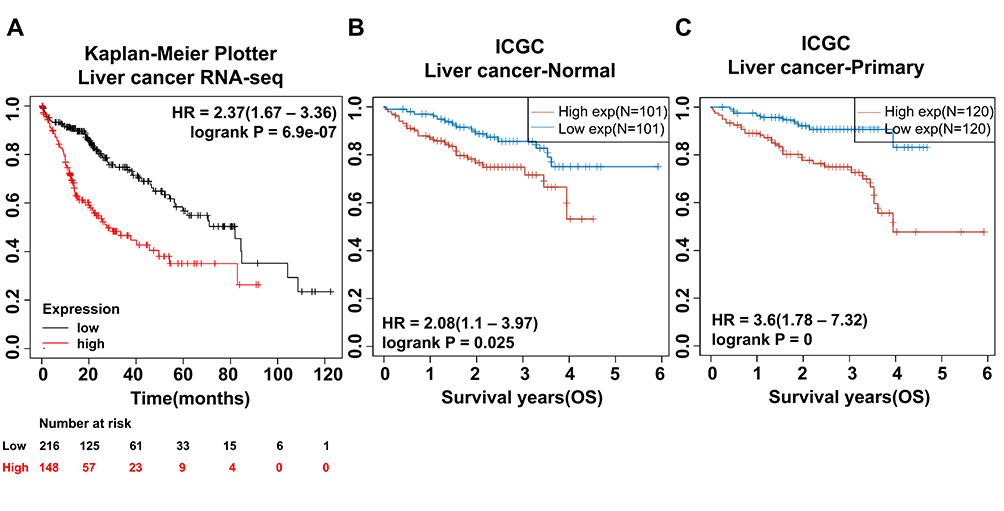


**Supplementary Figure 5.** (A) Liver cancer patients with high CBX3 expression have worse OS prognosis. (Kaplan-Meier Plotter). (B) Normal liver cancer patients with high CBX3 expression have worse OS prognosis (ICGC). (C) Primary liver cancer patients with high CBX3 expression have worse OS prognosis (ICGC).


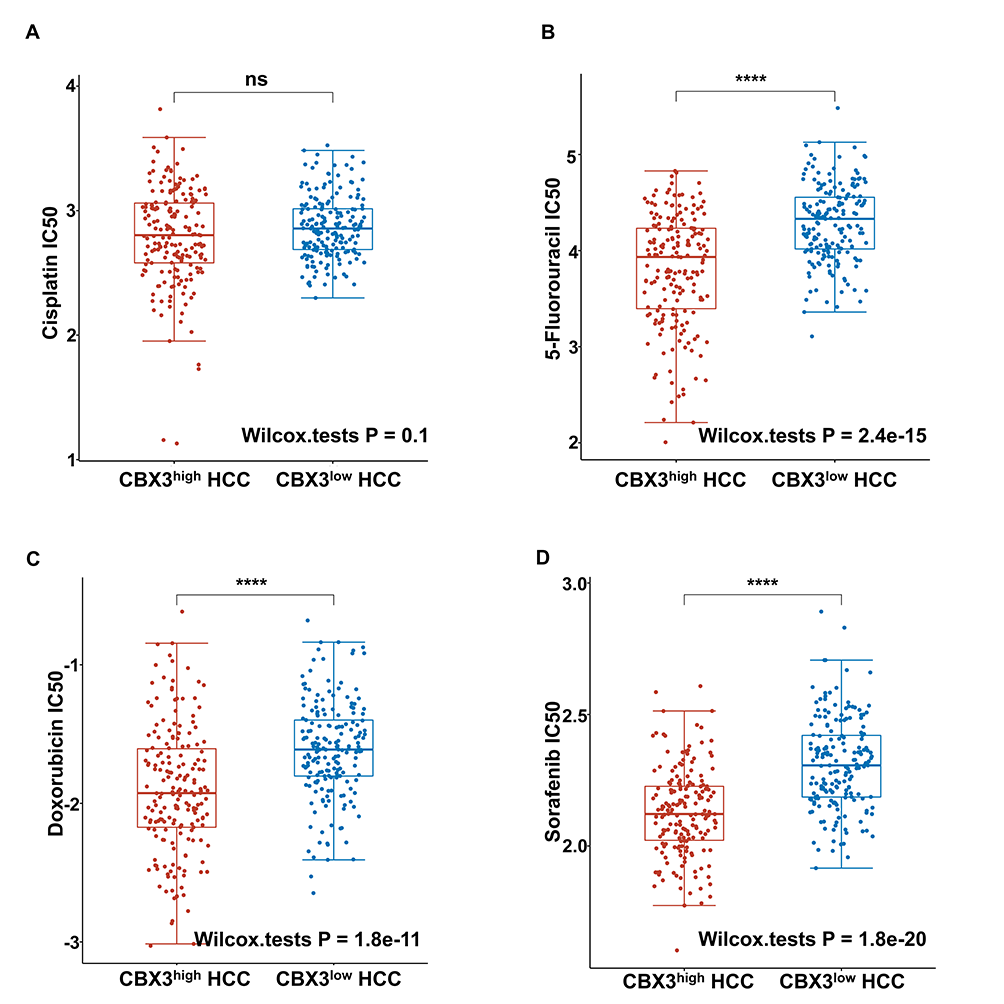


**Supplementary Figure 6.** In the GDSC website, the sensitivity of CBX3^high^ and CBX3^low^ HCC patients to (A) Cisplatin, (B) 5-Fluorouracil, (C) Doxorubicin and (D) Sorafenib were shown.
